# The genetics and pathology of mitochondrial disease

**DOI:** 10.1002/path.4809

**Published:** 2016-11-02

**Authors:** Charlotte L Alston, Mariana C Rocha, Nichola Z Lax, Doug M Turnbull, Robert W Taylor

**Affiliations:** ^1^ Wellcome Trust Centre for Mitochondrial Research, Institute of Neuroscience, The Medical SchoolNewcastle UniversityNewcastle upon TyneUK

**Keywords:** mitochondria, mitochondrial disease, mtDNA, respiratory chain deficiency, genetic diagnosis, muscle pathology, immunohistochemistry, neuropathology

## Abstract

Mitochondria are double‐membrane‐bound organelles that are present in all nucleated eukaryotic cells and are responsible for the production of cellular energy in the form of ATP. Mitochondrial function is under dual genetic control – the 16.6‐kb mitochondrial genome, with only 37 genes, and the nuclear genome, which encodes the remaining ∼1300 proteins of the mitoproteome. Mitochondrial dysfunction can arise because of defects in either mitochondrial DNA or nuclear mitochondrial genes, and can present in childhood or adulthood in association with vast clinical heterogeneity, with symptoms affecting a single organ or tissue, or multisystem involvement. There is no cure for mitochondrial disease for the vast majority of mitochondrial disease patients, and a genetic diagnosis is therefore crucial for genetic counselling and recurrence risk calculation, and can impact on the clinical management of affected patients. Next‐generation sequencing strategies are proving pivotal in the discovery of new disease genes and the diagnosis of clinically affected patients; mutations in >250 genes have now been shown to cause mitochondrial disease, and the biochemical, histochemical, immunocytochemical and neuropathological characterization of these patients has led to improved diagnostic testing strategies and novel diagnostic techniques. This review focuses on the current genetic landscape associated with mitochondrial disease, before focusing on advances in studying associated mitochondrial pathology in two, clinically relevant organs – skeletal muscle and brain. © 2016 The Authors. *The Journal of Pathology* published by John Wiley & Sons Ltd on behalf of Pathological Society of Great Britain and Ireland.

## Introduction

Mitochondria are double‐membrane‐bound organelles present in all nucleated eukaryotic cells, and are responsible for numerous cellular processes, including calcium homeostasis, iron–sulphur cluster biogenesis, apoptosis, and the production of cellular energy (ATP) by oxidative phosphorylation (OXPHOS) 1, 2. With bacterial origins, a historical symbiotic relationship evolved during which mitochondria became normal constituents of eukaryotic cells 3. Their ancestry remains apparent from their own multicopy genetic material [mitochondrial DNA (mtDNA)], with copy number varying greatly between individuals and across different tissues from the same individual. The 16.6‐kb circular mtDNA molecule encodes 13 subunits of the OXPHOS components, 22 mitochondrial tRNAs, and two subunits of the mitoribosomes 4. Additionally, the mitoproteome requires a further ∼1300 nuclear‐encoded proteins for producing, assembling or supporting the five multimeric OXPHOS complexes (I–V) and ancillary mitochondrial processes 5. It stands to reason that mitochondrial dysfunction can result from either mtDNA or nuclear gene defects, and can occur as a primary, congenital condition or a secondary, age‐associated effect attributable to somatic mutation 6.

The umbrella term ‘mitochondrial disease’ refers to a clinically heterogeneous group of primary mitochondrial disorders in which the tissues and organs that are most often affected are those with the highest energy demands. Clinical symptoms can arise in childhood or later in life, and can affect one organ in isolation or be multisystemic 7; the minimum disease prevalence in adults is ∼12.5 per 100 000 8, and ∼4.7 per 100 000 in children 9. There is a general lack of genotype–phenotype correlations in many mitochondrial disorders, which means that establishing a genetic diagnosis can be a complicated process, and remains elusive for many patients. This review provides a concise update on three areas where there have been major advances in our understanding in recent years 10, i.e. the molecular genetics, muscle pathology and neuropathology associated with mitochondrial disease, highlighting the range of new techniques that are improving the diagnosis of patients with suspected mitochondrial disease, with the aim of providing options to families at risk of an otherwise incurable condition.

## The genetics of mitochondrial disease

### Mitochondrial disease caused by mtDNA

Unlike nuclear DNA, which is diploid and follows Mendelian laws of inheritance, mtDNA is exclusively maternally inherited 11. The multicopy nature of mtDNA gives rise to heteroplasmy, a unique aspect of mtDNA‐associated genetics that occurs when there is coexistence of a mix of mutant and wild‐type mtDNA molecules (heteroplasmy). In contrast, homoplasmy occurs when all of the mtDNA molecules have the same genotype. Heteroplasmic mutations often have a variable threshold, i.e. a level to which the cell can tolerate defective mtDNA molecules 12. When the mutation load exceeds this threshold, metabolic dysfunction and associated clinical symptoms occur. Point mutations and large‐scale mtDNA deletions represent the two most common causes of primary mtDNA disease, the former usually being maternally inherited, and the latter typically arising *de novo* during embryonic development.

#### mtDNA point mutations

mtDNA point mutations (including small indel mutations) constitute a significant cause of human disease, with an estimated population prevalence of one in 200 13. Mutations have been reported in every mtDNA gene, and have been associated with clinical symptoms ranging from non‐syndromic sensorineural deafness to MELAS, a devastating syndromic neurological condition whose predominant features, i.e. mitochondrial encephalopathy, lactic acidosis, and stroke‐like episodes, give rise to the acronym. Clinical symptoms can present in child or adulthood, and mutations can be inherited (∼75% cases) or occur *de novo* (∼25% cases) 14. Maternally transmitted mtDNA defects may involve a clinically unaffected mother who harbours the familial mtDNA mutation below the threshold required for cellular dysfunction, although her oocytes harbour varying mutation loads, owing to the selection pressures of the mitochondrial bottleneck 15. It is therefore almost impossible to predict the recurrence risk for subsequent pregnancies, although prenatal testing of embryonic tissues by the use of chorionic villus biopsy or amniocentesis can provide an accurate measure of mtDNA heteroplasmy in the fetus, which can inform reproductive choices 16. The recurrence risk of *de novo* mtDNA point mutations is very low, except for the risk of germline mosaicism in maternal oocytes 14.

#### Single, large‐scale mtDNA deletions

Single, large‐scale mtDNA deletions have a population frequency of 1.5/100 000 8, with three main associated phenotypes: chronic progressive external ophthalmoplegia (PEO) (∼65% of cases), Kearns–Sayre syndrome (KSS) (∼30% of cases), and Pearson syndrome (<5% of cases) 17. Pearson syndrome is the most severe presentation associated with single, large‐scale mtDNA deletions; patients present early in life with sideroblastic anaemia and pancreatic dysfunction, and the condition is often fatal in infancy 18. KSS patients present before the of age 20 years with ptosis and/or PEO and pigmentary retinopathy, and may have multisystem involvement, including myopathy, ataxia, or cardiac conduction defects 17. PEO is the more benign presentation attributable to single mtDNA deletions, and is associated with ophthalmoplegia, ptosis, and myopathy 19. Unlike nuclear gene rearrangements, single, large‐scale mtDNA deletions often arise sporadically during embryonic development and have a low recurrence risk 20. Clinically affected women who harbour a large‐scale mtDNA deletion have a low (<10%) risk of transmission 20, and prenatal testing is informative for at‐risk pregnancies 16.

#### Secondary mtDNA mutations

Large‐scale mtDNA deletions and point mutations represent primary mtDNA defects, but secondary defects are other common causes of mitochondrial disease. Defective mtDNA maintenance, transcription, or protein translation, or a defective ancillary process such as mitochondrial import, can cause either quantitative (depletion of mtDNA copy number) or qualitative (affecting mtDNA genome integrity, resulting in multiple large mtDNA deletions) effects. These result from mutations affecting nuclear genes, and inheritance occurs in a Mendelian (or *de novo*) fashion.

### Mitochondrial disease caused by nuclear mitochondrial genes

The majority of the genes encoding the mitoproteome are in the nuclear genome 5 and follow Mendelian inheritance patterns. *De novo*, X‐linked, dominant and recessive inheritance cases have been reported in the literature 21, 22, 23, 24. The first nuclear mitochondrial gene mutation was identified in 1995 in *SDHA*, encoding a structural subunit of complex II 25, and there has been monumental progress in the discovery of mitochondrial disease candidate genes since then. New proteomic and transcriptomic approaches are being applied to models of human disease to uncover new candidates 26, 27, and patient analyses are validating their involvement in human pathology 28. The traditional approach of linkage analysis by the use of multiple affected family members has given way to massively parallel sequencing strategies, including whole exome sequencing (WES), either of affected singletons or of proband–parent trios, and new disease genes are still emerging over 20 years later. Of the ∼1300 proteins in the mitoproteome, mutations have been reported in >250 genes 29, and both new genes and new mechanisms involving genes already implicated in human disease through alternative pathways are being reported 30. It is apparent that more severe clinical phenotypes are often associated with recessive defects, presumably because of varying heteroplasmy levels in clinically affected tissues and the dichotomous effect of recessive mutations; therefore, mtDNA mutations are more common in adults, whereas nuclear gene defects are overrepresented in paediatric cases 31.

In this review, we delineate the nuclear mitochondrial disease genes into those that cause isolated and those that cause multiple respiratory chain complex deficiencies, for simplicity and brevity.

### Mitochondrial disease caused by nuclear mitochondrial genes: isolated respiratory chain complex deficiencies

**Figure 1:**
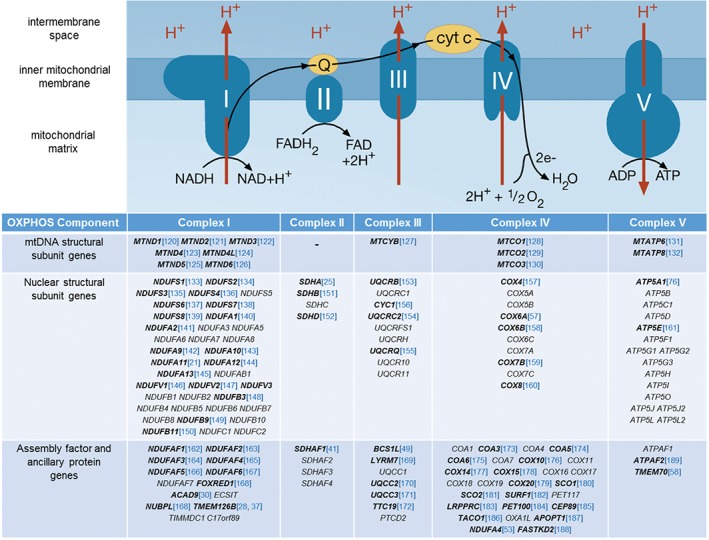
Schematic of the OXPHOS complexes, their component subunits, and associated ancillary factors. Multimeric protein complexes I–IV shuttle electrons along the respiratory chain, facilitated by the reduction of the cofactors coenzyme Q_10_ (Q) and cytochrome c (cyt c). Electron transfer is coupled to the transfer of protons (H^+^) across the inner mitochondrial membrane to generate a proton motive force, which is used by complex V (ATP synthase) to synthesize ATP. Characterization of OXPHOS complexes has identified the constitutive subunits that are either mtDNA‐encoded or nuclear‐encoded, and many of the nuclear‐encoded proteins involved in complex assembly, biogenesis, or ancillary function; genes in which mutations have been identified are shown in bold, and the first report of disease‐causing mutations is shown in blue.

Histochemical and biochemical evidence of an isolated respiratory chain complex deficiency can be suggestive of a mutation affecting either a structural subunit or an assembly/ancillary factor of one of the five OXPHOS complexes. Our current knowledge of the structural subunits and ancillary factors for each complex is summarized in Figure 1.

#### Isolated complex I deficiency

Complex I (NADH dehydrogenase) is composed of 44 structural subunits (seven of which are mtDNA‐encoded) with at least 14 ancillary/assembly factors 32, 33. Isolated complex I deficiency represents the biochemical phenotype for ∼30% of paediatric patients 34, of whom 70–80% have a nuclear gene defect 35. The clinical symptoms associated with complex I deficiency are heterogeneous, although the prognosis is typically poor, with rapid progression. Lactic acidosis is a common feature, although it is often present with other symptoms, such as cardiomyopathy or leukodystrophy. Mutations have been identified in 19 of the 37 structural subunits, and in 10 of 14 identified assembly factors. Although there are a few exceptions, such as the p.Trp22Arg *NDUFB3* 36 and p.Gly212Val *TMEM126B* European founder mutations 28, 37, and the p.Cys115Tyr *NDUFS6* Caucasus Jewish founder mutation 38, studies have revealed the majority of complex I deficiency mutations to be private and non‐recurrent 39. *NDUFS2* and *ACAD9* mutations account for a significant proportion of diagnoses, although it is likely that clearer genetic diagnostic trends will emerge from large diagnostic next‐generation sequencing (NGS) datasets 40.

#### Isolated complex II deficiency

Succinate dehydrogenase (SDH), unlike any of the other complexes of the mitochondrial OXPHOS system, is entirely nuclear‐encoded, and is involved in both the tricarboxylic acid cycle (where it metabolizes succinate to fumarate) and the respiratory chain (transferring electrons from FADH_2_ to reduce ubiquinone to ubiquinol). Complex II deficiency is rare (2–8% of mitochondrial disease cases 41, 42), with <50 patients having been reported. Biallelic mutations have been associated with congenital metabolic presentations, predominantly affecting either the central nervous system (CNS) or heart (hypertrophic cardiomyopathy, leukodystrophy, Leigh syndrome, and encephalopathy) 43, whereas heterozygous mutations are associated with cancer susceptibility, particularly pheochromocytoma and paraganglioma 44. Although SDH was initially believed to have distinct genotype–phenotype relationships (*SDHA* and *SDHAF1* being linked to mitochondrial disease, and *SDHB*/*SDHC*/*SDHD*/*SDHAF2* being linked with cancer susceptibility), it is emerging that there is phenotypic overlap, prompting tumour surveillance of unaffected relatives heterozygous for *SDHx* mutations 45, 46.

#### Isolated complex III deficiency

Ubiquinol–cytochrome *c* oxidoreductase, complex III of the respiratory chain, functions as a homodimer to transfer electrons from ubiquinol to cytochrome *b*, and then to cytochrome *c*. Complex III is composed of 11 structural subunits plus two heme groups and the Rieske iron–sulphur protein. Exercise intolerance is the clinical phenotype reported for >50% of patients with mutations in the mtDNA *MTCYB* gene, but cardiomyopathy and encephalomyopathy have also been noted 47. Pathogenic mutations have been reported in four of the nuclear‐encoded structural subunits plus five assembly/ancillary factors 48, with presentations including developmental delay, encephalopathy, lactic acidosis, liver dysfunction, renal tubulopathy, and muscle weakness 48, 49.

#### Isolated complex IV deficiency

Cytochrome *c* oxidase (COX), complex IV of the respiratory chain, is embedded in the inner mitochondrial membrane, and functions as a dimer, with two copper‐binding sites, two heme groups, one magnesium ion, and one zinc ion 50. Complex IV pumps protons across the inner mitochondrial membrane, contributing to the proton motive force for ATP synthase exploitation, and donates electrons to oxygen at the respiratory chain termini to form water. Complex IV has 13 structural subunits, and at least 26 additional proteins involved in assembly and biogenesis 51. *NDUFA4* was originally described as a complex I subunit gene, but has since been reassigned to complex IV, following functional studies 52 supported by the presence of *NDUFA4* defects in a patient with severe COX deficiency 53. Mutations have been reported in structural COX subunits, but most defects affect biogenesis/assembly proteins. Some proteins are linked tightly with specific aspects of COX biogenesis (e.g. COA6, involved in copper‐dependent COX2 biogenesis 54), and others have more diverse roles 55. Clinically, presentations are often early onset and devastating, predominantly affecting the heart and CNS (e.g. *SURF1*, in which >80 different mutations have been reported to cause Leigh syndrome 56), although a milder Charcot–Marie–Tooth phenotype has been associated with biallelic *COX6A1* variants 57.

#### Isolated complex V deficiency

ATP synthase, complex V, is the multimeric molecular motor that drives ATP production through phosphorylation of ADP. Utilizing the proton motive force generated by electron transport and proton pumping by the respiratory chain, the 600‐kDa complex consists of 13 different subunits (some of which have different isoforms; for example, *ATP5G1*, *ATP5G2* and *ATP5G3* encode subunit c isoforms), and involves at least three ancillary factors. Defects have been reported in only four nuclear complex V genes to date, with varied clinical phenotypes. The most common defects involve *TMEM70*, including a Roma *TMEM70* founder mutation causing lactic acidosis and cardiomyopathy 58, although encephalopathy and cataracts have been reported in other populations 59.

### Mitochondrial disease caused by nuclear mitochondrial genes: multiple respiratory chain defects

Mitochondrial function is regulated and maintained by ∼1300 nuclear genes; these nuclear genes are translated by cytosolic translational machinery, and the 5′ mitochondrial targeting sequence directs transport of the translated proteins into the mitochondrion, where they are required for diverse functions. These include the transcription of mitochondrial mRNA (e.g. *POLRMT* 60), mitochondrial DNA maintenance (e.g. *POLG* 61), regulation of mitochondrial dNTP pools (e.g. *RRM2B* 62), cellular signalling (e.g. *SIRT1* 63), and the translation of mtDNA‐derived proteins. Numerous subgroups of proteins are involved in mitochondrial gene translation: mitochondrial aminoacyl tRNA synthetases, which are responsible for charging each mitochondrial tRNA molecule with the appropriate amino acid (e.g. *AARS2* 64), proteins involved in RNA processing (e.g. *MTPAP* 65), mitoribosomal proteins (e.g. *MRPL44* 66), and proteins involved in mitochondrial tRNA modification (e.g. *TRMU* 67). Defects in ≥250 nuclear mitochondrial genes have now been reported in association with multiple respiratory chain defects and clinical mitochondrial disease 29. The genetic diagnostic pathway for these disorders is complex, and WES is often the most successful strategy 68.

### Non‐OXPHOS mitochondrial disease

Not all mitochondrial disease patients have evidence of respiratory chain enzyme dysfunction, but have other evidence of mitochondrial disease, such as elevated lactate levels, suggestive magnetic resonance imgaing brain changes, and multisystem involvement. Genetic causes include defective enzymes of the Krebs cycle (e.g. aconitase/*ACO2* 69) or cofactor transport (e.g. thiamine transporter/*SLC19A3* 70).

## Molecular genetic analysis of mitochondrial disease

**Figure 2:**
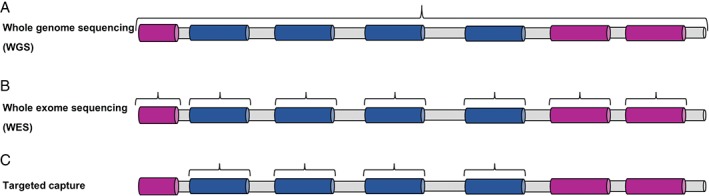
NGS strategies employed in the genetic diagnosis of mitochondrial disease. (A) WGS analyses all coding and non‐coding regions of the genome. (B) WES targets only the coding exons plus immediate intron–exon boundaries. (C) Target capture facilitates sequencing of a predetermined genomic region or list of candidate disease genes. Non‐coding/intronic regions are shaded grey, exons of candidate genes are shaded blue, and exons of non‐candidate genes are shaded pink.

In the absence of effective treatments, provision of a firm genetic diagnosis facilitates genetic counselling and access to reproductive options for patients and their families. Given the small size of the mtDNA genome, this is often sequenced in suspected mitochondrial disease patients to exclude a primary mtDNA defect before nuclear genes are scrutinized. NGS‐based testing is becoming more prevalent 71, and also provides an accurate measure of mtDNA heteroplasmy. NGS technologies are revolutionizing the genetic testing pipeline in the diagnostic genetic laboratory, with Sanger sequencing of candidate genes on a sequential basis being replaced with powerful, high‐throughput analysis. A variety of options are currently being implemented – targeted panels of candidate genes 36, unbiased WES 72, and whole genome sequencing (WGS) 73 (Figure 2). Custom, panel‐based NGS strategies can be very successful in providing a rapid genetic diagnosis in the clinical setting, but this success depends on the degree of characterization to ensure that the appropriate candidate genes are targeted. Stratification according to respiratory chain defect can be appropriate for many patients in whom muscle biopsy is available, but even then it may be misleading – a number of patients with an isolated complex I deficiency have, in fact, a defect of mitochondrial translation 40; moreover, this strategy can be ineffective for genes that show inconsistent biochemical profiles 74. Stratification according to clinical phenotype is similarly complicated by genetic heterogeneity 75.

Despite a proven track record in a research setting and the increasing availability of affordable NGS options to diagnostic laboratories, the case has yet to be made regarding the clinical validity of unrestricted WES within a diagnostic setting. One solution to the stratification dilemma, and one that has been successfully implemented for the analysis of other heterogeneous Mendelian disorders, is a combination of unbiased WES with targeted analysis of ‘virtual’ gene panels 76, 77; this allows informative reporting of negative results, and removes the possibility of incidental findings. Further analysis of the WES data for patients lacking a diagnosis following virtual panel analysis could be subsequently undertaken in a research setting. Indeed, most of the candidate genes included in diagnostic virtual panels have their origins in research. WES has been incredibly fruitful in elucidating genes involved in human pathology, including heterogeneous mitochondrial clinical phenotypes such as cardiomyopathy, with mutations identified in AARS2 78, MRPL3 79, MTO1 80, and ACAD9 72. New candidate genes continue to be discovered in a research setting, and are then included in diagnostic screening; one success is exemplified by the report of patients harbouring mutations in TMEM126B, a candidate gene identified by research‐based complexome profiling 27, 28, 37. Similarly, characterization of predicted mitochondrial proteins of unknown function is another critical strategy for identifying novel disease candidate genes 26.

## Investigating muscle pathology associated with mitochondrial disease

As discussed above, the laboratory investigation of suspected mitochondrial disease is complex, and algorithms employ a multidisciplinary approach using clinical and functional studies to guide genetic analysis 81. Although mitochondrial disorders are characterized by a wide spectrum of clinical presentations, owing to the high metabolic requirements, muscle is frequently affected – either exclusively (e.g. myopathy and chronic progressive external ophthalmoplegia) or as a predominant feature in multisystem phenotypes 81, 82. In both scenarios, muscle involvement can arise from mutations in nuclear or mtDNA genes, and the association with distinctive histopathological hallmarks makes muscle an excellent postmitotic surrogate for the study of many multisystem mitochondrial disorders. Diagnostic centres specializing in mitochondrial disorders employ numerous techniques to assess mitochondrial function, including the assessment of individual mitochondrial OXPHOS activities in vitro 83. Although useful for identifying widespread mitochondrial defects, this technique has some limitations; it requires large quantities of muscle (typically 50–100 mg of tissue) and may fail to detect subtle OXPHOS deficiencies, especially when only a few muscle fibres are affected (e.g. mild mosaic deficiencies). Furthermore, only complexes I–IV can be reliably assessed in frozen muscle.

**Figure 3:**
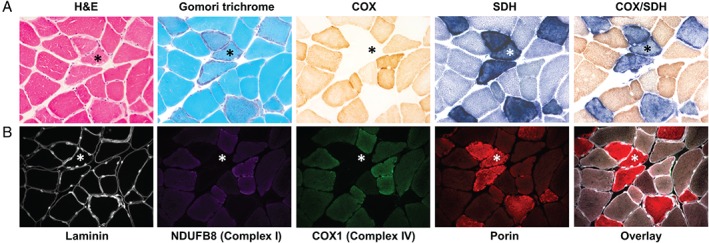
Histological, histochemical and immunohistochemical hallmarks of mitochondrial pathology in primary mtDNA‐related disease. (A) Serial skeletal muscle (vastus lateralis) sections from a patient with a single, large‐scale mtDNA deletion were stained with H&E and modified Gomori trichrome to assess basic muscle morphology and the presence of RRFs, respectively. The individual COX, SDH and sequential COX/SDH histochemical reactions show fibres manifesting mitochondrial accumulation and focal COX deficiency. (B) The lack of histochemical assays to assess other OXPHOS complex activities prompted the development of a quadruple immunofluorescence assay that can quantify the levels of complex I (NDUFB8 subunit), complex IV (COX1 subunit), laminin, and a mitochondrial mass marker (porin), all within a single 10‐µm section. A highlighted COX‐deficient fibre (*) shows focal accumulation of sarcolemmal mitochondria around the periphery of the fibre, and downregulated expression of both complex I and IV proteins. (All images taken at ×20 objective magnification.)

The histological and histochemical examination of serially‐sectioned muscle can provide crucial evidence of mitochondrial pathology. Haematoxylin and eosin (H&E) and modified Gomori trichrome stains assess basic muscle morphology, providing information on fibre size and the presence of any abnormal inclusions or central nuclei which are indicative of muscle denervation (Figure 3). The modified Gomori trichrome stain 84, 85 specifically highlights connective tissue (light blue), muscle fibres (blue), and mitochondria (red), and allows the detection of ragged‐red fibres (RRFs) 86. RRFs are characterized by a ‘fibre cracking’ appearance and abnormal subsarcolemmal proliferation of mitochondria, resulting from a compensatory response to a respiratory chain biochemical defect 87. RRFs can show either normal oxidative enzyme activities (often reported in association with the m.3243A>G mutation or some sporadic MTCYB mutations) or COX deficiency associated with a wide range of mtDNA‐related genetic disorders 88. They represent a characteristic histopathological feature of mitochondrial disorders, however, they are not entirely diagnostic, as they are also seen with normal ageing 6 and other muscle conditions 89, 90.

**Figure 4:**
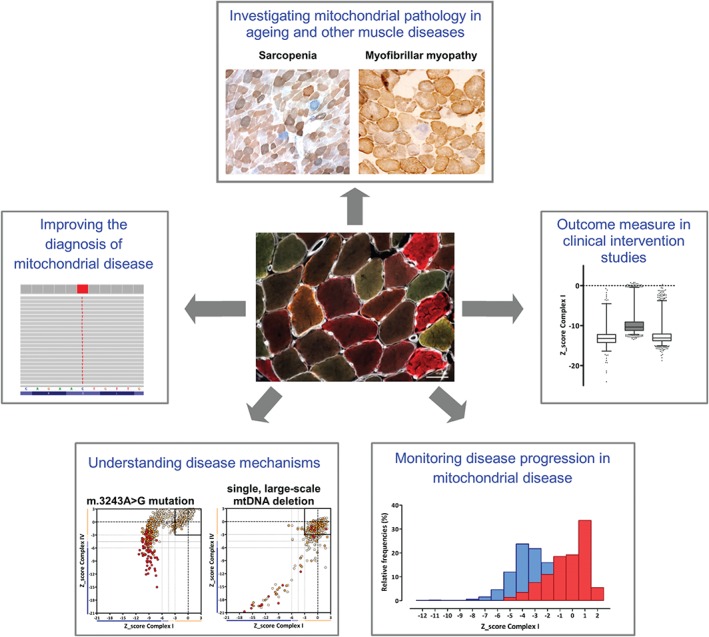
Current and future applications of a quantitative, quadruple OXPHOS immunofluorescence assay. Given its capacity to interrogate levels of both complex I and IV – and additional OXPHOS components – at a single muscle fibre level, we believe that the quadruple immunofluorescence assay can be applied to several areas of diagnostic and research activity in the laboratory to help investigate the role of mitochondrial biochemical defects 96. We are already implementing this methodology in a diagnostic setting, validating the assay with biopsies from patients showing a range of mtDNA‐related and nuclear genetic diagnoses of mitochondrial disease. The assay also shows promise as a powerful tool with which to investigate the mitochondrial pathological changes observed in ageing and other myopathies (e.g. myofibrillar myopathies 90), to investigate molecular disease mechanisms and mitochondrial disease progression, as well as providing an extremely sensitive outcome measure in clinical therapeutic intervention studies (e.g. pharmacological agents or exercise) aimed at improving muscle oxidative capacity in patients with mitochondrial disease.

Sequential COX/SDH histochemistry is the standard method used to assess mitochondrial respiratory chain function in muscle cryosections 91, 92, the activities of the partially mtDNA‐encoded complex IV (COX), and the fully nuclear‐encoded complex II (SDH). By combining both reactions in a single slide (Figure 3A, COX/SDH panel), fibres or cells with mitochondrial dysfunction are easily identifiable, and are seen as a mosaic reduction or loss of COX activity with preserved SDH activity (blue fibres), indicative of an underlying mtDNA‐related abnormality 93, 94. The absence of routine histochemical assays to evaluate other OXPHOS complexes, such as complex I, which is the largest and most commonly affected OXPHOS complex in mitochondrial disorders 95, has prompted the recent development of a novel high‐throughput immunofluorescence assay to fill the gap in the diagnostic repertoire 96. This technique enables accurate quantification of the two most commonly affected OXPHOS components, namely complexes I and IV 97, together with a mitochondrial mass marker (porin) in individual muscle fibres on a single 10‐µm tissue section (Figure 3B). The semi‐automatic quantification of a large number of muscle fibres is facilitated by labelling laminin to define fibre boundaries. Image analysis is exclusively based on intensity measurements, increasing its accuracy and reliability, and is automated (http://iah‐rdevext.ncl.ac.uk/immuno/). We are currently optimizing the immunodetection of antibodies to assess complex III and complex V, in order to better quantify the full extent of mitochondrial respiratory deficiency in patient muscle sections, but the opportunity to assess this at a single‐fibre level shows great potential for both diagnostic and research applications (Figure 4).

## Neuropathology associated with mitochondrial disease

Neurological symptoms are particularly common, and may be devastating in patients with mitochondrial disease, including sensorineural deafness, cerebellar ataxia, peripheral neuropathy, dementia, and epilepsy 81. In recent years, a number of neuropathological studies have documented the characteristic features of neurodegeneration in patients with mitochondrial disease, and these have spurred the development of novel tools with which to understand the mechanisms underlying neural dysfunction and cell death.

### New insights into mechanisms of neurodegeneration

**Figure 5:**
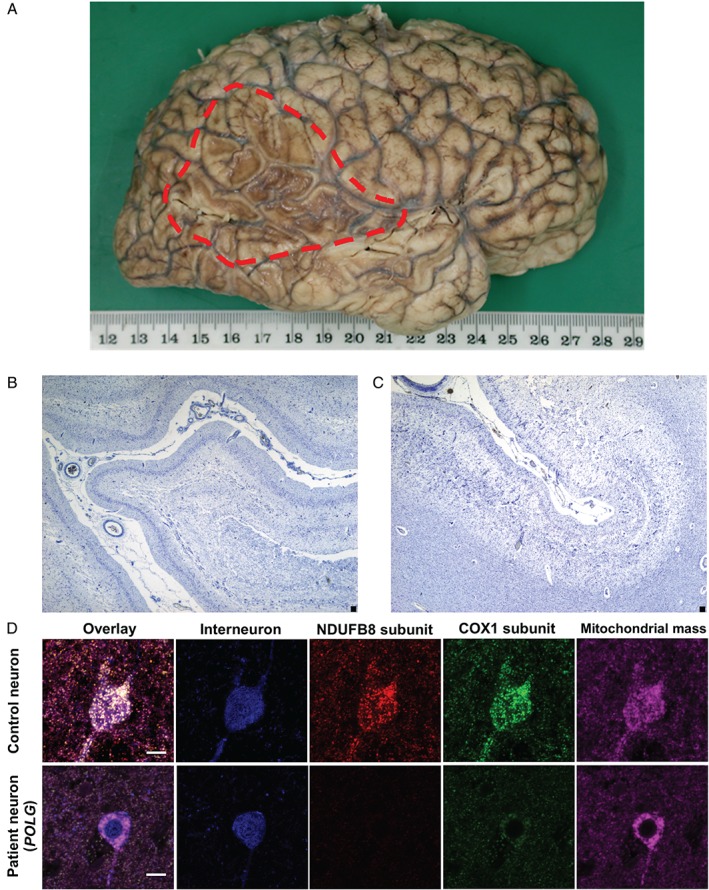
Neuropathological changes associated with stroke‐like episodes in patients with mitochondrial disease. (A) Extensive cortical necrosis affecting the occipital, temporal and parietal lobes in a brain from a patient harbouring the m.3243A>G mutation. (B, C) Microscopic analysis reveals atrophy, microvacuolation and severe neuronal loss in the frontal cortex of a patient with the m.3243A>G mutation [(B) Cresyl fast violet staining] and in the temporal cortex of a patient with the m.8344A>G mutation [(C) Cresyl fast violet staining]. (D) Respiratory chain abnormalities include downregulation of subunits constituting complex I (red; NDUFB8 subunit) and complex IV (green; COXI) relative to intact mitochondrial mass (magenta; porin) in inhibitory interneurons (blue; GAD 65–67) in a patient harbouring autosomal recessive POLG mutations. Scale bar: 10 µm.

Upon neuropathological investigation, the brains from patients with mitochondrial disease often show atrophy, cortical lesions, evidence of neuronal cell loss, and mitochondrial OXPHOS abnormalities in the remaining cells. Patients with the heteroplasmic m.3243A>G mutation and a MELAS phenotype often develop foci of cortical necrosis on the surface of the brain (Figure 5A). These are often referred to as ischaemic‐like lesions, as they resemble stroke penumbra but do not conform to a particular vascular territory. It is proposed that these lesions evolve during stroke‐like episodes, and may be initiated by mitochondrial respiratory abnormalities in neurons that act to alter the balance of excitation and inhibition in neural networks, promoting neuronal hyperexcitability 98. This is important, as seizures are frequently detected by electroencephalography in patients who have had a stroke‐like episode 99. Although focal necrotic changes associated with the m.3243A>G mutation have been commonly documented, it is important to note that patients harbouring other genetic defects (e.g. the m.8344A>G mutation 100 and autosomal recessive *POLG* mutations 101, 102) also develop cortical lesions, suggesting shared mechanisms underpinning their formation. These lesions typically affect posterior brain regions, including the occipital, parietal and temporal lobes, and feature microvacuolation and neuronal cell dropout (Figure 5B, C), neuronal eosinophilia, astrogliosis, and secondary myelin loss. Recent studies have proposed that vulnerability of GABAergic interneurons could underpin neuronal hyperexcitability, as dramatic downregulation of OXPHOS subunits constituting complexes I and IV has been observed within interneurons (Figure 5D) 103; other theories suggest that aggregation of abnormally enlarged mitochondria and the presence of mitochondrial respiratory chain abnormalities in the cerebral microvasculature may contribute to impaired cerebral perfusion 104, 105. Although the precise mechanisms are not known, the emergence of lesions in the brain reflect an acute process leading to rapid neuronal loss that can occur on the background of more chronic and protracted cell loss throughout the brain.

**Figure 6:**
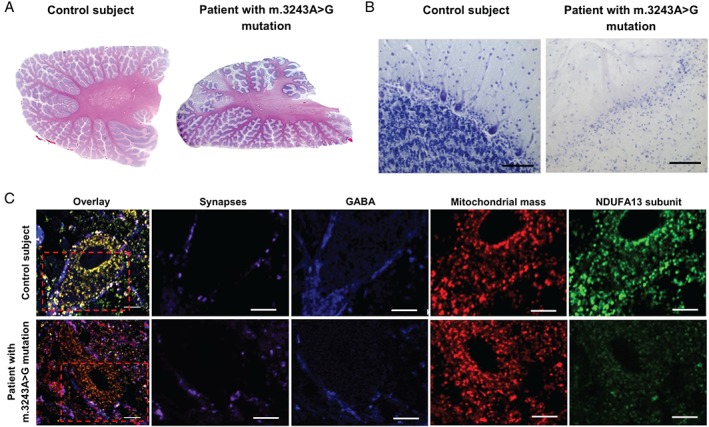
Cerebellar pathology in patients with the m.3243A>G mutation. (A) Numerous areas of necrosis are evident throughout the cerebellar cortex of a patient in comparison with control cerebellum (H&E staining). (B) Extreme neuronal loss is seen microscopically, affecting Purkinje cells and granule cells in the cortex (Cresyl fast violet staining). Scale bar: 100 µm). (C) In dentate nucleus neurons and in GABAergic (blue; GAD 65–67) synapses (magenta; synaptophysin) from Purkinje cells, there is downregulation of complex I (green; NDUFA13) relative to mitochondrial mass (red; COX4I2). Scale bar: 10 µm).

The cerebellum is frequently involved in mitochondrial disease, with many patients developing cerebellar ataxia. Neuropathologically, the cerebellum reveals signs of lesions (Figure 6A) similar to those observed in the cortex, global Purkinje cell dropout (Figure 6B), and loss of dentate nucleus neurons 106. Recent work has shown downregulation of protein subunits constituting complex I in remaining Purkinje cells, their GABAergic synapses, and dentate nucleus neurons (Figure 6C). In conjunction, there is evidence of neuronal network remodelling with thickened dendritic arborizations, axonal torpedoes, and altered synaptic density 107, 108, 109. There is a distinct lack of correlation between the severity of cell loss and the heteroplasmy level of mutated mtDNA in surviving neurons, suggesting that other factors must be important in determining cell loss 110.

Patients harbouring a single large‐scale mtDNA deletion may develop KSS, which is associated with severe demyelination and spongiosis of the white matter tracts of the brain, including the cerebrum, cerebellum, spinal cord, and brainstem 111. The loss of myelin is proposed to be attributable to specific vulnerability of mature oligodendrocytes, the myelin‐producing glia, where a loss of respiratory chain activity resulting from the mtDNA deletion causes a distal oligodendrogliopathy and subsequent loss of myelin products 112. It is not known why the mtDNA deletion preferentially affects oligodendrocytes.

In summary, neuropathological studies have shown that neuronal cell loss can occur via two different processes: an acute event, such as in stroke‐like lesions, or a global, protracted loss of cells. There is no evidence of protein accumulation within neurons, surviving neurons frequently show respiratory chain deficiency, including downregulation of complex I subunits, and there is a lack of correlation of cell loss and mtDNA heteroplasmy in remaining neurons.

### Tools to aid the study of mitochondrial neuropathology

Recently, a number of novel methods have been developed to provide further insights into potential mechanisms of neurodegeneration, particularly for understanding the early events leading to irreversible neuronal cell loss. Clear lipid‐exchanged acrylamide‐hybridized rigid imaging/immunostaining/*in situ*‐hybridization‐compatible tissue hydrogel has paved the way for large volumes of archived, postmortem material to be investigated with three‐dimensional analysis of the neuronal networks 113. This will enable a greater understanding of neuronal vulnerability in mitochondrial disease 114. The recent development of induced pluripotent stem cell technology allows the cellular transfection of human patient fibroblasts with four key transcription factors to confer pluripotency. These pluripotent cells can subsequently be differentiated into neurons and glial cells, and the effects of both the nuclear genome and mitochondrial genome can be investigated to determine disease mechanisms, efficacy of drug treatment, and cell replacement therapies 115, 116. Additionally, a number of transgenic mouse models utilizing Cre/Lox technology to selectively knock out nuclear mitochondrial genes within specific populations of neurons and glial cells are promising for the understanding of specific disease mechanisms 117, 118, 119.

## Challenges for the future

Developing an effective treatment for mitochondrial disease is an enormous challenge that is dependent on the integration of clinical understanding of disease progression, molecular genetic mechanisms, and neuropathological features in mitochondrial disease. Patient‐based clinical, molecular genetic and histopathology studies can then inform the development of appropriate disease model systems to determine mechanisms and treatment to ultimately improve the lives of patients with mitochondrial disease.

## Author contributions statement

All authors contributed to the drafting of the manuscript and its critical revision for important intellectual content.
